# DIC Score in Pregnant Women – A Population Based Modification of the International Society on Thrombosis and Hemostasis Score

**DOI:** 10.1371/journal.pone.0093240

**Published:** 2014-04-11

**Authors:** Offer Erez, Lena Novack, Ruthy Beer-Weisel, Doron Dukler, Fernanda Press, Alexander Zlotnik, Nandor Gabor Than, Aaron Tomer, Moshe Mazor

**Affiliations:** 1 Department of Obstetrics and Gynecology, Soroka University Medical Center, School of Medicine, Faculty of Health Sciences, Ben Gurion University of the Negev, Beer Sheva, Israel; 2 Department of Epidemiology, School of Medicine, Faculty of Health Sciences, Ben Gurion University of the Negev, Beer Sheva, Israel; 3 Department of Anesthesiology and Intensive Care, Soroka University Medical Center, School of Medicine, Faculty of Health Sciences, Ben Gurion University of the Negev, Beer Sheva, Israel; 4 Department of Obstetrics and Gynecology, School of Medicine, Wayne State University, Detroit, Michigan, United States of America; 5 Blood Bank and Transfusion Medicine, Soroka University Medical Center, School of Medicine, Faculty of Health Sciences, Ben Gurion University of the Negev, Beer Sheva, Israel; Maastricht University Medical Center, Netherlands

## Abstract

**Objectives:**

The objectives of this study were: 1) To determine the component needed to generate a validated DIC score during pregnancy. 2) To validate such scoring system in the identification of patients with clinical diagnosis of DIC.

**Material and Methods:**

This is a population based retrospective study, including all women who gave birth at the ‘Soroka University Medical Center’ during the study period, and have had blood coagulation tests including complete blood cell count, prothrombin time (PT)(seconds), partial thromboplastin time (aPTT), fibrinogen, and D-dimers. Nomograms for pregnancy were established, and DIC score was constructed based on ROC curve analyses.

**Results:**

1) maternal plasma fibrinogen concentrations increased during pregnancy; 2) maternal platelet count decreased gradually during gestation; 3) the PT and PTT values did not change with advancing gestation; 4) PT difference had an area under the curve (AUC) of 0.96 (p<0.001), and a PT difference ≥1.55 had an 87% sensitivity and 90% specificity for the diagnosis of DIC; 5) the platelet count had an AUC of 0.87 (p<0.001), an 86% sensitivity and 71% specificity for the diagnosis of DIC; 6) fibrinogen concentrations had an AUC of 0.95 (p<0.001) and a cutoff point ≤3.9 g/L had a sensitivity of 87% and a specificity of 92% for the development of DIC; and 7) The pregnancy adjusted DIC score had an AUC of 0.975 (p<0.001) and at a cutoff point of ≥26 had a sensitivity of 88%, a specificity of 96%, a LR(+) of 22 and a LR(−) of 0.125 for the diagnosis of DIC.

**Conclusion:**

We could establish a sensitive and specific pregnancy adjusted DIC score. The positive likelihood ratio of this score suggests that a patient with a score of ≥26 has a high probability to have DIC.

## Introduction

The process of labor and delivery is associated with an increased risk for severe maternal hemorrhage [1]. Therefore, as an adaptive physiologic mechanism, pregnancy is associated with a physiologic prothrombotic state [2], [3] resulting in increased thrombin generation locally and systemically. Sufficient local hemostasis is achieved by the abundance of tissue factor in the decidua [4], [5], chorionic membranes and amniotic fluid [6]–[8]. In addition, systemic changes are observed in the maternal plasma including : 1) increased concentrations of clotting factors VII, VIII, IX, X and XII [3], [9]–[13] and fibrinogen; 2) a reduction in the concentration of anticoagulant proteins such as protein S and tissue factor pathway inhibitor (TFPI)-1 [14]–[18]; 3) acquired resistance to activated protein C sensitivity [18]–[20]; and 4) reduced fibrinolysis as a result of low activation of plasminogen activator inhibitor (PAI) I and II [13], [21]–[25].

In spite of these physiologic changes in maternal hemostasis, uncontrolled peripartum bleeding, resulting in consumption coagulopathy and disseminated intravascular coagulation (DIC), is one of the leading causes for maternal mortality worldwide [26]. Although DIC results from a wide spread activation of both clotting and fibrinolysis systems leading to: 1) systemic production of fibrin split products, and thrombi that leads to end-organ ischemia; 2) increased vascular permeability due to activation of the kinin system; and 3) microangiopathic hemolysis, during pregnancy hemorrhage is the leading mechanisms for the development DIC. The most prevalent etiologies for such bleeding are post-partum hemorrhage, placental abruption, placenta previa, uterine rupture, cervical and vaginal lacerations, as well as infection [27]. In modern obstetrics, the development of advanced pharmacological and surgical techniques to control bleeding, as well as the availability of advance transfusion services are the major factors that led to the substantial reduction in maternal mortality as a result of hemorrhage in developed countries. Nevertheless, severe peri-partum bleeding is still a leading cause for maternal morbidity and mortality even in these countries [26], [27]. Currently, aside a clinical assessment, there are no effective tools to identify patients with acute bleeding at risk for DIC.

The International Society for Thrombosis and Hemostasis has adopted a score that assists in the diagnosis and the identification of patients at risk for the development of DIC [28]. This score is based on readily available coagulation assays including PT, PTT, fibrinogen and D-dimer or fibrin split products. In non-pregnant patients, there is a good correlation between an abnormal score result and the development of DIC [28]. However, in light of the physiologic changes of the coagulation cascade during gestation, this score could not be implemented in pregnant women. On the other hand, the morbidity and mortality associated with severe hemorrhage and consumption coagulopathy leading to DIC during pregnancy emphasizes the need for the adjustment of this ISTH DIC score to these patients. Therefore, the objectives of this study were: 1) to determine the component needed to generate a validated DIC score during pregnancy; and 2) to validate a new scoring system for the identification of patients with clinical DIC;

## Materials and Methods

### Study population

This is a population based retrospective study, including all women who gave birth at the ‘Soroka University Medical Center’ during the study period, and have had blood coagulation tests including complete blood cell count, prothrombin time (PT)(seconds), partial thromboplastin time (aPTT)(seconds), fibrinogen (g/L), and D-dimers (mg/L).

Exclusion criteria included: multiple gestation, chromosomal abnormalities or structural defects of the fetus. The use of the database was possible as the ‘Soroka’ University Medical Center is a tertiary medical center, which exclusively serves the population of the Negev, and all deliveries of the region takes place in its labor and delivery suites. The Department of Obstetrics and Gynecology has a computerized database of all the deliveries, the information is captured from the patients' medical records and coded according to the ICD-9 diagnosis into the database by trained secretaries. The information of all the laboratory results was incorporated into the patient's file in the computerized database.

There were 19,889 women who met the inclusion criteria and had 24,693 deliveries; 87 deliveries were complicated with DIC and comprised the study group; the rest (n = 24,606) comprised the comparison group. The diagnosis of DIC in the data base was according to ICD -9 code 776.2. The coding of DIC in the database was based upon the clinical diagnosis reported in the medical records. The clinical diagnosis of DIC at the Soroka University Medical Center in based upon severe maternal hemorrhage associated with prolonged PT as well as PTT, and low fibrinogen concentrations that required blood products transfusion.

The study was approved by the Soroka University Medical Center institutional review board

### Outcome variables

In order to construct our DIC score, we performed several steps of analysis: 1) Calculating the 5^th^, 50^th^, and 95^th^ percentiles of the platelet count (×10^3^/µL), fibrinogen concentrations (g/L), prothrombin time (PT)(seconds), partial thromboplastin time (aPTT)(seconds), and D-dimer according to gestational age at sample collection. 2) Generating the cutoff values based on Receiver Operator Characteristic curves of each analyte with the chance to develop DIC including platelet count (×10^9^/L), fibrinogen concentrations (g/L), and PT difference defined as the difference between the result of the patient and that of the laboratory normal control. 3) Constructing a multiple logistic regression model in which the cutoff values were derived from the ROC curves analysis and clinically relevant values were tested for their association with the development of DIC. 4) Building a DIC scoring system and comparing its diagnostic values to those of the existing International Society on Thrombosis and Hemostasis score.

### Clinical definitions

*Please see* Supplementary information S1 in File S1.

### Statistical analysis

Continuous variables were presented as mean ± standard deviation (SD) and compared by t-test, ANOVA and Wilcoxon rank-sum test depending on their distribution. Categorical variables were presented as proportion out of available observations and compared between groups using Chi-square test and Fisher's exact test.

To build a DIC score, the contribution of each of the analytes tested was assessed using a log-binomial regression predicting the DIC diagnosis. We used robust estimates of standard errors in order to adjust for multiple deliveries of the same women. The model was built based on 75% of the study population that were used as the training sample and the remaining 25% were used for validation. The weight of each of the analytes in the resulting DIC score was determined based on the relative risk (RR) effect that was estimated by regression, whereas the analyte with the minimal impact was assigned as the reference value of “1” and weights of the rest of the factors represented their relative effect, i.e. RR of a factor divided by RR of a minimal factor.

Goodness-of-fit of the DIC score was assessed by the Area-Under-the Curve (AUC) of the Receiver Operator Curve (ROC) calculated on the training, and validated on the validation samples. As a part of a sensitivity analysis, the resultant DIC score was calculated within deliveries at risk (defined by preeclampsia, or post-partum hemorrhage or ante-partum death of a fetus) and within deliveries with abruption of placenta. P-value of less than 0.05 was considered significant. Analysis was done by SAS package (SAS Institute Inc, Cary, NC, USA).

## Results

### Study population

The rate of DIC in the study cohort was 0.35% (87/24,693) of all deliveries. Pregnancies complicated with DIC included older women, and those were more likely to be grand multiparous or to have infertility treatments (Table 1). The leading pregnancy complications associated with DIC were the following: placental abruption (49.4%), post-partum hemorrhage (29.9%), severe preeclampsia (12.6%), and uterine rupture (5.7%) (Table 1). The most prominent neonatal outcomes associated with DIC were lower mean birth weight, small for gestational age neonates, preterm birth, and increased total perinatal mortality (DIC group: 44% vs. comparison group: 4%, p<0.001)(Table 2).

**Table 1:** Maternal Characteristics - by DIC Diagnosis.

Maternal Characteristics	Comparison group N = 24,606 Deliveries	DIC group N = 87 Deliveries	P-value
Age, years (n)	29.6±6.2 (24579)	31.9±6.1 (87)	<.001
Jewish Origin	52.2% (12846/24606)	37.9% (33/87)	0.01
Gravidity			
1st Pregnancy	25.5% (6280/24601)	8.0% (7/87)	<0.001
2–5 Pregnancies	50.1% (12320/24601)	41.4% (36/87)	
6+ Pregnancies	24.4% (6001/24601)	50.6% (44/87)	
Parity			
1st Delivery	33.3% (7764/23325)	13.2% (10/76)	<0.001
2–5 Deliveries	53.0% (12372/23325)	60.5% (46/76)	
6+ Deliveries	13.7% (3189/23325)	26.3% (20/76)	
Infertility Treatments	7.7% (1888/24606)	13.8% (12/87)	0.04
Chronic Hypertension	5.7% (1402/24606)	0.0% (0/87)	0.01
GDM Class A	6.5% (1598/24606)	3.4% (3/87)	0.38
GDM Class B-R	2.4% (601/24606)	0.0% (0/87)	0.28
Severe Preeclampsia	5.3% (1312/24606)	12.6% (11/87)	0.01
Mild Preeclampsia	14.0% (3433/24606)	3.4% (3/87)	0.003
Abruption of Placenta	2.6% (641/24606)	49.4% (43/87)	<0.001
Uterine Rupture	0.2% (56/24606)	5.7% (5/87)	<0.001
Post-Partum Hemorrhage	2.6% (646/24606)	29.9% (26/87)	<0.001

GDM – gestational diabetes mellitus.

**Table 2:** Neonatal Characteristics - by DIC Diagnosis.

Newborn Characteristics	No DIC Diagnosis N = 25,573 Newborns	DIC Diagnosis N = 91 Newborns	P-value
Birthweight, gr Mean±SD (N)	2953.3±748.4 (25564)	2376.3±972.6 (91)	<.001
Male gender	51.9% (13279/25573)	51.6% (47/91)	1.0
Small for Gestational Age	7.5% (1922/25573)	14.3% (13/91)	0.03
Large for Gestational Age	9.1% (2329/25573)	6.6% (6/91)	0.58
Weight			
<1,500gr	5.4% (1376/25564)	22.0% (20/91)	<0.001
1,500–2,500gr	16.0% (4096/25564)	26.4% (24/91)	
>2,500gr	78.6% (20092/25564)	51.6% (47/91)	
Gestational Age, wk			
<28	2.1% (532/25573)	12.1% (11/91)	<0.001
28–32	3.0% (773/25573)	13.2% (12/91)	
32–34	3.0% (774/25573)	2.2% (2/91)	
34–37	12.1% (3093/25573)	24.2% (22/91)	
37+	79.8% (20401/25573)	48.4% (44/91)	
APD	2.5% (629/25573)	30.8% (28/91)	<0.001
IPD	0.1% (35/25573)	3.3% (3/91)	<.001
PPD	1.4% (365/25573)	9.9% (9/91)	<.001
Total perinatal mortality	4.0% (1027/25573)	44.0% (40/91)	<.001

APD- ante-partum death; IPD – intra-partum death; PPD – post-partum death; wk- weeks; and gr- grams.

### Changes in the results of the diagnostic tests with advancing gestation

**Figure 1:**
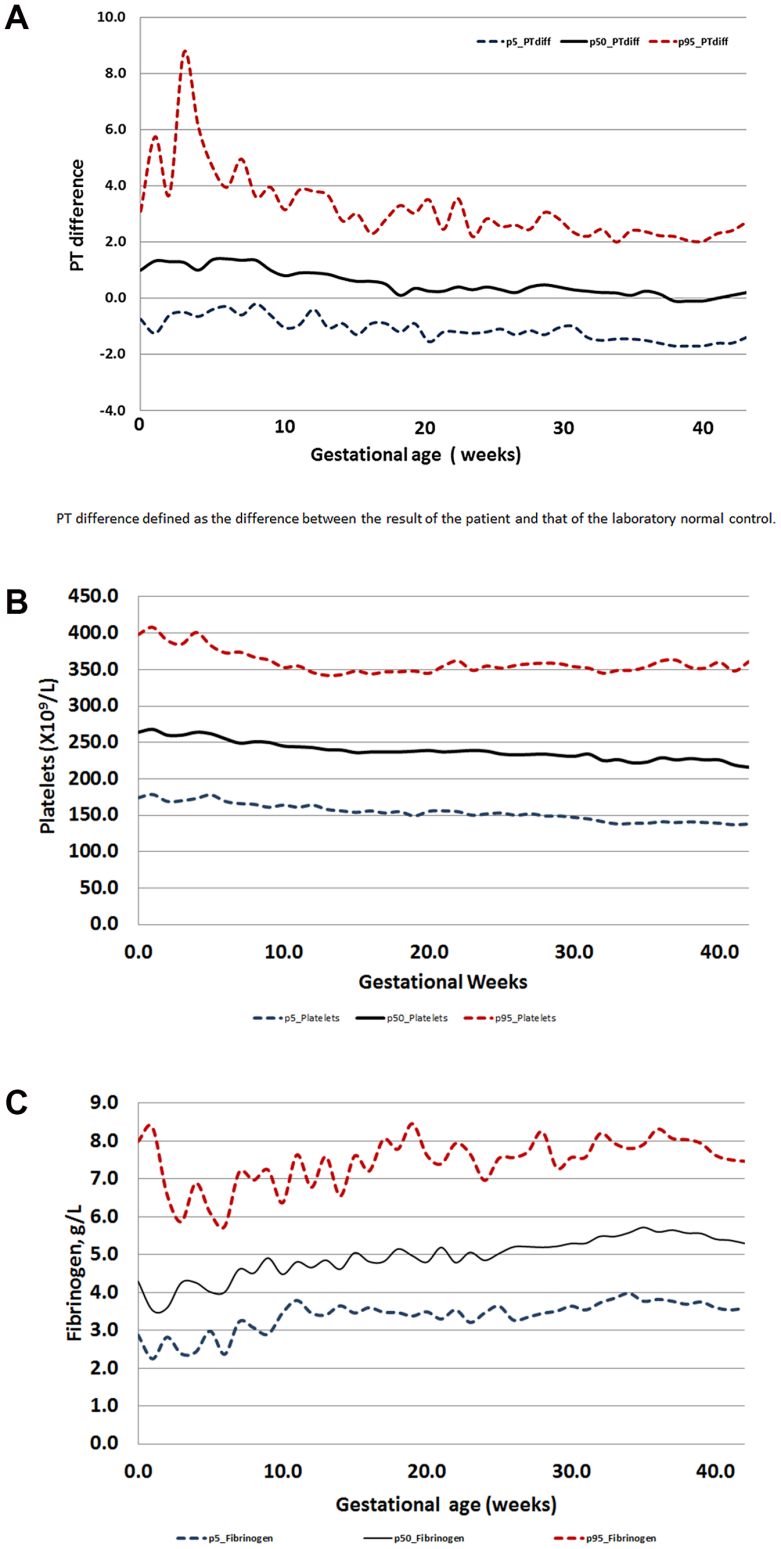
The changes in the major components of the pregnancy modified DIC score: a)- PT- difference; b)- platelets; and c)- fibrinogen, with advancing gestations.

The PT difference decreased during pregnancy (Figure 1a), the maternal plasma platelet count decreased throughout gestation (Figure 1b), while the maternal plasma fibrinogen concentrations increased during pregnancy (Figure 1c). The mean and the percentiles of the PT difference, the platelets, and fibrinogen concentrations according to gestational age are presented in Tables S1–3 in File S1, respectively.

### The association between maternal plasma concentrations and the PT difference with the development of DIC

**Figure 2:**
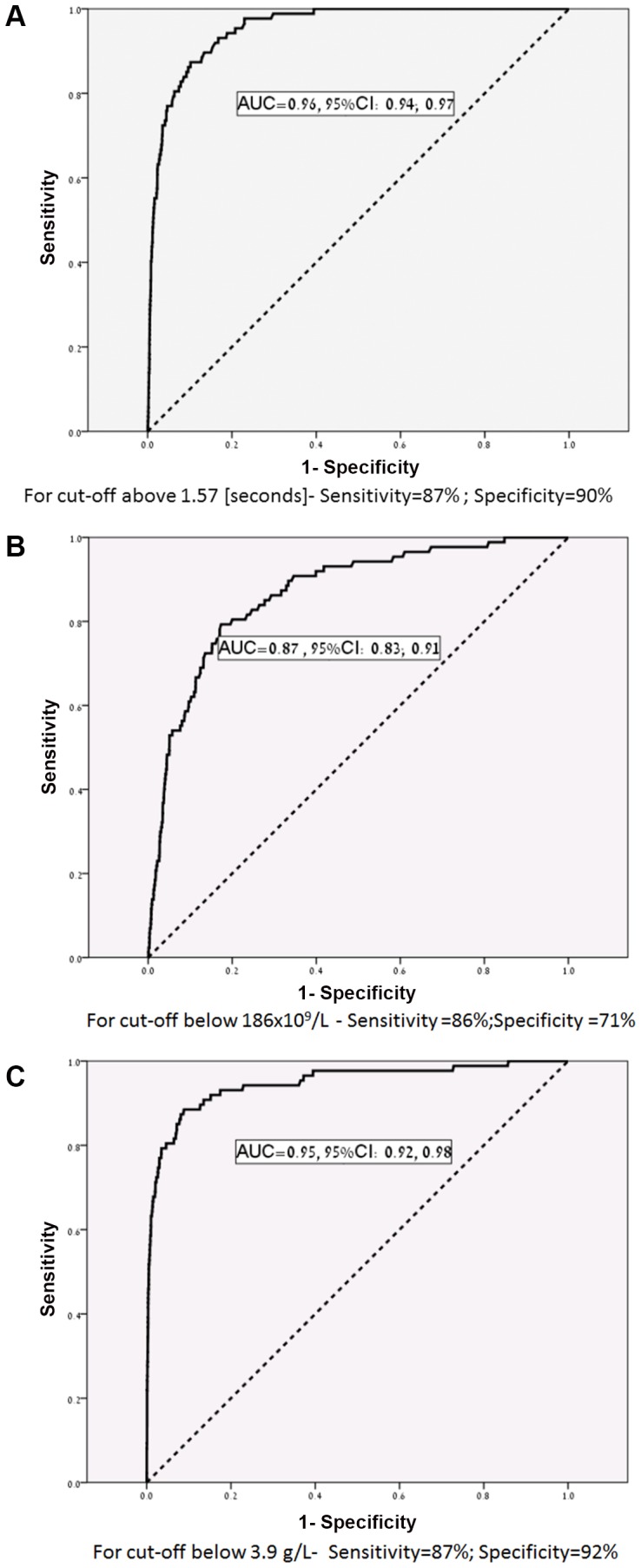
ROC curve analysis for the association of the major components of the pregnancy modified DIC score: a)- PT- difference; b)- platelets; and c)- fibrinogen, with the development of DIC.

We performed ROC Curves analysis to determine the diagnostic value of each analyte near the development of DIC. The PT difference had an area under the curve (AUC) of 0.96 (p<0.001), whereas a PT difference above 1.55 yielded a sensitivity of 87% and specificity of 90% for the diagnosis of DIC (Figure 2a). The maternal plasma platelet count was significantly associated with the development of DIC and had an AUC of 0.87 (p<0.001), with platelet count of ≤186 X ×10^3^/µL provided with sensitivity of 86% and a specificity of 71% for the diagnosis of DIC (Figure 2b). The maternal plasma fibrinogen concentrations were significantly associated with the development of DIC, and had an AUC = 0.95 (p<0.001) and a cutoff point ≤3.9 g/L characterized by a sensitivity of 87% and a specificity of 92% for the development of DIC (Figure 2c).

**Figure 3:**
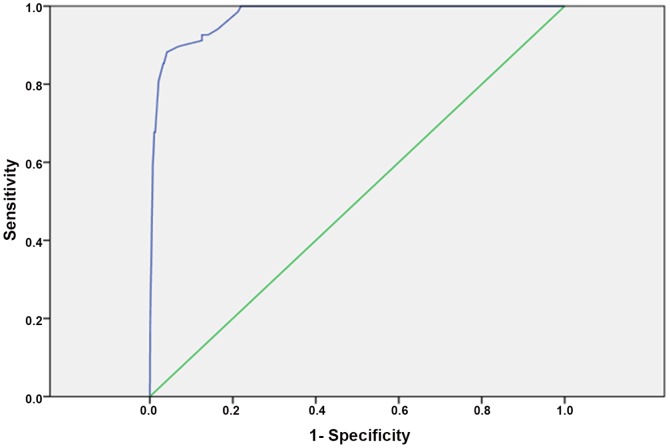
ROC curve analysis for the association of the modified DIC score with the development of DIC.

The log-binomial regression analysis for the association of the different analytes according to the cutoff generated from the ROC curves analysis for the development of DIC is presented in Table 3. A PT difference of beyond 1.5 presented the highest risk for the development of DIC [RR 60.3, 95% confidence interval (CI) 6.9–525.6], followed by fibrinogen below 3.0 g/L (RR 59.0 95% CI 20.7–168.7), and platelet count of <50 X ×10^3^/µL had an adjusted RR of 3.1 95% CI (2.8–272.9). According to the results of the log-binomial regression we composed a score that is presented in Table 3 which takes into account the relative contribution of each analyte to the diagnosis of DIC. The resultant DIC score was 1 for half of the study population and 6.1 on average, with minimal and maximal values 0 and 52, respectively. The modified DIC score was then tested in its diagnostic value for DIC and the ROC analysis yielded an area under the curve of 0.98 (p<0.001) and at a cutoff point of ≥26 had a sensitivity of 88% and a specificity of 96%, this indices are better than the results of the individual analytes (Figure 3). The cutoff point of the score = 26 was based on the maximal Youden statistic calculation. At a cutoff point of ≥26 the pregnancy adjusted DIC score had a positive likelihood ratio of 22 and a negative likelihood ratio of 0.125. Analysis of DIC in the validation samples (25% of the study population) yielded similar results with area under the curve equal 0.97.

**Table 3:** An effect of components of the new DIC score – results of logistic regression.

	Effect of individual analytes		Effect of individual analytes adjusted to other tests		Assigned Weight^1^
	Relative Risk	p-value	Relative Risk	p-value	
PT difference (seconds)					
<0.5	1.0		1.0		0
0.5–1	12.7	0.031	29.3	<0.001	5
1.0–1.5	27.7	0.005	68.8	<0.001	12
>1.5	60.3	<0.001	558.1	<0.001	25
Platelets (10^9^/L)					
<50	3.1	0.06	89.2	<0.001	1
50–100	5.2	<0.001	56.2	<0.001	2
100–185	2.9	0.001	12.8	<0.001	1
>185	1.0		1.0		0
Fibrinogen (g/L)					
<3.0	59.0	<0.001	662.9	<0.001	25
3.0–4.0	13.4	<0.001	59.1	<0.001	6
4.0–4.5	2.4	0.320	6.8	0.03	1
>4.5	1.00		1.0		0

1 Weight was calculated as relative risk of each of the adjusted factors to the relative risk of a factor with minimal effect.

### Sensitivity Analysis

When we included in the model only patients with abruption, preeclampsia, and post-partum hemorrhage the area under the curve of the ROC analysis was 0.969(p<0.001) and a cutoff point of ≥26 had a sensitivity of 85.4% and a specificity of 96.8%, this indices are better than the results of the individual analytes (Figure 3). At a cutoff point of ≥26 the pregnancy adjusted DIC score had a positive likelihood ratio of 26.7 and a negative likelihood ratio of 0.15. The area under the curve for patients diagnosed with abruption of placenta was 0.97 (p<0.001) and the DIC score at or above 26 yielded sensitivity of 93% and specificity of 90.5%, with corresponding negative and positive likelihood ratios equal 9.79 and 0.08.

### The comparisons of the performance of the obstetric DIC score with the current DIC scores available

In order to further validate our results, the performance of the DIC score we have developed were compared to that of a modified version of the DIC score adopted by the International Society of Thrombosis and Hemostasis (ISTH) (we have excluded the D-Dimer from the score). Due to the differences in patient selection and definition we could not compare our score to that proposed by Terao in 2007 [29].

Since abruption was the most prevalent cause for blood transfusion and DIC in our population, we used these patients for the comparison between the DIC scores. Out of 684 women with abruption, 150 (21.93%) needed blood transfusion and 43 (6.29%) had DIC. The first comparison was in the ability to identify patients with abruption who needed blood and blood product transfusion. Our DIC score at a cutoff point of 26 had an area under the curve of  = 0.98; 95%CI: (0.96; 0.99), a sensitivity of 88% and a specificity of 96%. The modified ISTH score at a cutoff point of 0.5 had an AUC = 0.85; 95%CI: (0.78; 0.91), a sensitivity of 74%, and a specificity of 95%.

## Discussion

### Principal findings of the study

1) pregnancy is associated with significant changes in the major components of the ISTH overt DIC score; 2) by using only three components of this score, platelet count, fibrinogen concentrations and the PT difference, we were able to construct a pregnancy modified DIC score that had an area the curve of 0.975 (p<0.001), and at a cutoff of ≥26 points had a sensitivity of 88% and a specificity of 96% for the diagnosis of DIC; and 3) at this cutoff the pregnancy modified DIC score had a positive likelihood ratio score of 22 and a negative likelihood ratio score of 0.125.

### Why is a DIC score important?

DIC is a serious and life threatening complication that can result from several mechanisms, including acute and chronic consumptive coagulopathy, endothelial dysfunction and platelets activation, and acute liver dysfunction [28], [30]–[41]. The most prominent obstetrical pathologies associated with the development of DIC are post-partum hemorrhage, placental abruption, HELLP syndrome, preeclampsia, retained dead fetus, acute fatty liver, and septic abortion [42]–[50]. The effect of these pathologies on the coagulation profile of the patients and the risk to develop DIC is not evident in all cases [42]–[50]. In addition, there is no single laboratory or clinical test that is sensitive and specific enough to diagnose DIC. In light of the above three DIC scores were previously developed [28], [51], [52]. All these scores use simple and readily available coagulation tests including platelet count, PT elongation, fibrinogen and fibrin split products/D-dimer concentrations [28], [51], [52]. The three DIC scores currently in use include the following: 1) the Japanese Ministry of Health and Welfare (JMHW) score that was proposed in 1983 [51]; 2) the ISTH overt DIC score that was published in 2001 [28]; and 3) the Japanese Association for Acute Medicine (JAAM) score that was published in 2005 [52]. All these three use the same components to generate their scores, which have good predictive value for the diagnosis of DIC and the identification of critically ill non-pregnant patients that are about to die. These scores can be used not only as a diagnostic but also as prognostic tool. Thus, in the non-pregnant state a DIC score is important in the diagnosis of patients with DIC and carries a diagnostic and prognostic value [53]–[58].

Terao et al [29] suggested in 1987 an obstetrical DIC score based on 77 patients with DIC identified in 100 centers in Japan, of which their score identified 70 (90%). The score included three main categories: 1) etiology- stating whether there is a prominent etiology that can explain the development of DIC; 2) clinical manifestation- including bleeding and organ dyfunction; and 3) laboratory tests- including PT, fibrinogen, FDP, and platelets; a minimum score of ≥7 needed for the diagnosis of DIC. However, this score was not validated in comparison to the normal obstetric population, and it is currently not in wide clinical use [29].

### What are the changes in the components of the DIC score during pregnancy?

The ISTH overt DIC score is based on four components including platelet count, fibrinogen concentrations, PT difference (elongation of PT) and the concentrations of D-dimer or fibrin split products. Three of these parameters change during pregnancy [28]. Indeed, fibrinogen increases during gestation especially through the third trimester and declines only two days after delivery [59]–[64]. The underlying mechanisms leading to this change are not clear, is it a physiological change of pregnancy, or is it due to the fact that fibrinogen is an acute phase reactant, and its concentration reflect the changes in the maternal inflammatory status during gestation. An animal model suggests that the concentrations of fibrinogen are influenced by changes in estrogen concentrations which increase as labor approaches [65].

DIC is associated with a low platelet count, indeed thrombocytopenia is reported in up to 98% of patients with this condition [66], [67]. Moreover, about 50% of patients with DIC will have a platelet count lower than 50×10^6^/L [66], [67]. Pregnancy is a unique state in which the platelet count slightly decreases with advancing gestation [68]–[70], and about 7% of all pregnant women will suffer from thrombocytopenia [70]; moreover, it has been proposed that pregnancy is a compensated state of platelet consumption [69]. However, thrombocytopenia is a hallmark of severe pregnancy complications such as preeclampsia and hemolysis, elevated liver enzymes and low platelets (HELLP) syndrome [41], [49], [50], [71]–[76]. The latter is a leading cause for DIC during pregnancy [47], [49], [50]; and the severity of HELLP syndrome was defined by some according to the degree of the thrombocytopenia [41], [49], [50], [71]–[76], and women with HELLP syndrome who had a platelet count of <50×10^9^/L were at increased risk for DIC and liver hematoma/rupture [49], [50], [73], [74]. Indeed, in the modified score presented herein, thrombocytopenia of <50×10^9^/L platelets is a strong identifier of DIC in pregnant women.

The PT difference (the difference between the result of the patient and that of the laboratory normal control) is a crude marker for DIC [28]. Prolongation of PT suggests that the concentration of the coagulation factors is below 50%. Indeed, Chakraverty et al [77] reported that among 235 patients admitted to an adult intensive care unit, clinical coagulopathy, defined as bleeding unexplained by local or surgical factors, was identified in 13.6% of patients; moreover, a prothrombin time (PT) difference > or  = 1.5 was found in 66% of patients and a platelet count <100×10^9^/L in 38% of patients. Both factors were predictive of excessive bleeding and poor outcome [77]. Although during normal pregnancy the values of PT do not change substantially, the PT difference gives fast information regarding the status of the clotting factors. Indeed, in our modified score, a PT difference of >1.5 was associated with an adjusted relative risk of 558.1 95%CI 75.6–4120.8 to have DIC, and its assigned weight in the pregnancy modified DIC score was 25 points, while 26 points is the diagnostic cutoff for DIC.

The fourth component of the ISTH overt DIC score is the concentrations of D-dimer or fibrin split products [28]. These parameters are also increased in thromboembolic diseases, recent surgery, and inflammatory condition; thus, they can poorly differentiate these patients from those with DIC [78]. Moreover, Hatada et al [79] studied the cutoff values of fibrin related markers in the diagnosis of overt DIC. The authors reported that the use of fibrin related markers including D-dimer were useful in the diagnosis and prognosis of DIC resulting from infection [79]; however, it was less useful in the diagnosis of DIC resulting from solid or hematologic tumors [79]. During pregnancy, the concentrations of D-dimer or fibrin split products changes extensively, and it has been proposed that they have no diagnostic value during gestation [25], [63], [64]. Indeed, D-dimer concentration of 0.5 mg/L is considered as the upper limit of the normal value in non-pregnant patients [80]–[82]. However, during pregnancy, D-dimer level increases substantially after 20 weeks of gestation [63]; and during the third trimester practically all patients have a concentration >0.5 mg/L [25], [63], [64]. Therefore, the diagnostic value of this parameter is very low during pregnancy, and we, at the Soroka University Medical, hardly use the concentrations of D-dimer or fibrin split products in the clinical management of our pregnant patients. In light of the changes in the maternal concentrations of D-dimer during pregnancy, the lack of its clinical utility during gestation, and the small number of D-dimer tests performed at our medical center, this marker was not included in our score.

### What are differences between the modified DIC score and the ISTH overt DIC score?

We present here for the first time a DIC score that is specific to pregnancy. Our pregnancy modified DIC score has a high sensitivity and specificity to identify patients with DIC in the general obstetric population. The positive Likelihood Ratio score above 10 suggest a high probability that a positive test in our score will be really diagnostic for DIC. This is also correct for the negative Likelihood Ratio score suggesting that a negative result is true and the probability that such patient has DIC is very low.

The ISTH overt DIC score has proven to be both sensitive and specific for the diagnosis of DIC in non-pregnant patients [28]. Moreover, it was also associated with a good survival prediction of non-pregnant patients hospitalized in intensive care units [52], [83], [84]. Nevertheless, the reference values for the calculation of the ISTH score do not take into account the physiological changes that occur in these parameters during pregnancy. Indeed, when we used a modified version of this score according to data we have available (i.e. excluding D-dimer) the ISTH DIC score did not performed as well as it does in non-pregnant patients.

We agree with the approach presented by Terao and his colleagues [29] regarding the need for a predisposing event that puts the mother at risk for DIC as an essential condition for calculating the DIC score. However, we could not compare our results to their findings due to the large diversity in the definition of risk factors and score calculation.

Of note, our score is based on a retrospective study and analysis of an already established clinical database. In order to validate this score a large prospective clinical trial is needed. Moreover, this score will not be complete without the implementation of bedside point of care assays like thromboelstgram that can give an indication of the type of coagulopathy and the needed blood products to amend it.

### Strengths and weakness of our study

The major weakness of our study is its retrospective nature that carries the inherited limitation of working with an established dataset. Nevertheless, this is the largest cohort of pregnant patients with DIC published so far with a large reference population of women without DIC who had normal and complicated pregnancies.

### Conclusions

We present herein a novel DIC score for pregnant women. This score is sensitive as well as specific, and can serve clinicians worldwide. This pregnancy modified DIC score can be used to identify women who develop DIC even in Labor and Delivery departments that lack advance laboratories facilities.

## Supporting Information

File S1 Table S1, PT difference percentiles according to gestational age. Table S2, Platelets concentration (10^9^/L) percentiles according to gestational age. Table S3, Fibrinogen concentration percentiles according to gestational age. Supplementary information S1, clinical definitions.
(DOCX)
