# Supplementary material for: DIC Score in Pregnant Women – A Population Based Modification of the International Society on Thrombosis and Hemostasis Score
Source: PLoS One. 2014 Apr 11;9(4):e93240. doi: 10.1371/journal.pone.0093240 (PMC3984105; doi:10.1371/journal.pone.0093240)
Supplement: File S1 — Table S1, PT difference percentiles according to gestational age. Table S2, Platelets concentration (109/L) percentiles according to gestational age. Table S3, Fibrinogen concentration percentiles according to gestational age. Supplementary information S1, clinical definitions. (DOCX) [file pone.0093240.s001.docx]

**Supplementary Tables**

Supplementary Table 1- PT difference percentiles according to gestational age

| **Gestational age at sample collection (weeks )** | **Number of patients** | **Mean**  **PT difference** | **P-1** | **P-5** | **P-10** | **P-25** | **P-50** | **P-75** | **P-90** | **P-95** | **P-99** |
| --- | --- | --- | --- | --- | --- | --- | --- | --- | --- | --- | --- |
| 0 | 27 | -1.89 | -119.80 | -0.75 | -0.30 | 0.35 | 1.00 | 1.80 | 2.90 | 3.10 | 43.4 |
| 1 | 30 | 2.04 | -1.35 | -1.25 | -0.63 | 0.25 | 1.33 | 2.50 | 3.33 | 5.75 | 24.6 |
| 2 | 43 | 1.34884 | -1.05 | -0.60 | 0.00 | 0.35 | 1.30 | 2.25 | 2.56 | 3.70 | 4.4 |
| 3 | 58 | 1.87112 | -0.60 | -0.50 | -0.200 | 0.30 | 1.28 | 2.30 | 3.68 | 8.75 | 14.1 |
| 4 | 64 | 1.74141 | -3.35 | -0.65 | -0.400 | 0.50 | 1.00 | 1.75 | 3.70 | 6.10 | 25.5 |
| 5 | 74 | 1.59527 | -0.75 | -0.40 | -0.050 | 0.70 | 1.38 | 2.00 | 3.20 | 4.65 | 9.3 |
| 6 | 77 | 1.46916 | -0.50 | -0.30 | -0.100 | 0.75 | 1.40 | 2.10 | 3.45 | 3.95 | 4.9 |
| 7 | 79 | 1.70042 | -1.70 | -0.60 | -0.100 | 0.60 | 1.35 | 2.80 | 3.90 | 4.95 | 7.65 |
| 8 | 81 | 1.44167 | -2.90 | -0.20 | -0.050 | 0.75 | 1.35 | 2.10 | 2.90 | 3.60 | 5.25 |
| 9 | 73 | 1.39247 | -1.20 | -0.60 | -0.250 | 0.25 | 1.00 | 2.40 | 2.98 | 3.95 | 11.05 |
| 10 | 92 | 0.90870 | -1.85 | -1.05 | -0.500 | 0.03 | 0.80 | 1.73 | 2.55 | 3.15 | 5.00 |
| 11 | 90 | 1.24639 | -2.40 | -0.95 | -0.300 | 0.30 | 0.90 | 1.70 | 3.08 | 3.85 | 14.60 |
| 12 | 75 | 1.02200 | -0.80 | -0.40 | -0.200 | 0.10 | 0.90 | 1.50 | 3.10 | 3.80 | 4.30 |
| 13 | 85 | 0.86500 | -2.20 | -1.05 | -0.600 | -0.10 | 0.85 | 1.60 | 2.30 | 3.65 | 5.65 |
| 14 | 88 | 0.82216 | -1.40 | -0.90 | -0.600 | -0.10 | 0.70 | 1.38 | 2.20 | 2.75 | 9.35 |
| 15 | 74 | 0.79223 | -2.60 | -1.30 | -0.700 | -0.10 | 0.60 | 1.70 | 2.35 | 3.00 | 4.05 |
| 16 | 97 | 0.66082 | -1.35 | -0.90 | -0.600 | -0.20 | 0.60 | 1.20 | 1.85 | 2.30 | 5.70 |
| 17 | 116 | 0.64583 | -1.50 | -0.90 | -0.650 | -0.15 | 0.50 | 1.20 | 2.25 | 2.80 | 3.10 |
| 18 | 89 | 0.44101 | -1.70 | -1.20 | -0.800 | -0.50 | 0.10 | 0.90 | 2.10 | 3.30 | 7.10 |
| 19 | 90 | 0.52972 | -1.35 | -0.90 | -0.675 | -0.30 | 0.35 | 0.95 | 2.03 | 3.03 | 8.55 |
| 20 | 79 | 0.54705 | -2.65 | -1.55 | -0.900 | -0.40 | 0.25 | 1.05 | 2.80 | 3.50 | 9.10 |
| 21 | 105 | 0.54619 | -1.90 | -1.20 | -0.950 | -0.30 | 0.25 | 1.10 | 2.10 | 2.45 | 5.65 |
| 22 | 121 | 0.84063 | -2.00 | -1.20 | -0.600 | -0.10 | 0.40 | 1.60 | 2.50 | 3.55 | 6.65 |
| 23 | 201 | 0.39502 | -1.70 | -1.25 | -0.900 | -0.40 | 0.30 | 1.05 | 1.80 | 2.20 | 4.20 |
| 24 | 197 | 0.57961 | -2.30 | -1.20 | -0.750 | -0.30 | 0.40 | 1.20 | 2.10 | 2.83 | 4.38 |
| 25 | 189 | 0.44890 | -2.10 | -1.10 | -0.800 | -0.30 | 0.30 | 1.00 | 2.05 | 2.55 | 3.70 |
| 26 | 219 | 0.37310 | -1.90 | -1.30 | -0.900 | -0.40 | 0.20 | 1.00 | 1.90 | 2.60 | 3.90 |
| 27 | 274 | 0.63444 | -1.60 | -1.15 | -0.850 | -0.20 | 0.40 | 1.10 | 1.80 | 2.45 | 5.56 |
| 28 | 276 | 0.61415 | -2.35 | -1.30 | -0.900 | -0.28 | 0.48 | 1.30 | 1.95 | 3.05 | 4.87 |
| 29 | 287 | 0.53908 | -1.80 | -1.05 | -0.900 | -0.20 | 0.40 | 1.10 | 1.85 | 2.83 | 6.50 |
| 30 | 311 | 0.39741 | -1.80 | -1.00 | -0.800 | -0.30 | 0.30 | 1.00 | 1.70 | 2.35 | 3.70 |
| 31 | 336 | 0.37075 | -2.15 | -1.40 | -0.950 | -0.35 | 0.25 | 0.98 | 1.65 | 2.20 | 3.90 |
| 32 | 429 | 0.41311 | -2.10 | -1.50 | -1.100 | -0.45 | 0.20 | 0.90 | 1.80 | 2.45 | 6.60 |
| 33 | 424 | 0.22442 | -1.95 | -1.45 | -1.100 | -0.50 | 0.19 | 0.80 | 1.50 | 2.00 | 3.45 |
| 34 | 491 | 0.24520 | -2.10 | -1.45 | -1.100 | -0.55 | 0.10 | 0.85 | 1.65 | 2.40 | 3.80 |
| 35 | 536 | 0.33234 | -2.30 | -1.50 | -1.100 | -0.50 | 0.25 | 0.90 | 1.70 | 2.37 | 3.60 |
| 36 | 680 | 0.26475 | -2.40 | -1.60 | -1.250 | -0.65 | 0.15 | 0.80 | 1.67 | 2.23 | 6.95 |
| 37 | 983 | 0.05982 | -2.35 | -1.70 | -1.250 | -0.750 | -0.1000 | 0.650 | 1.550 | 2.20000 | 4.5625 |
| 38 | 1284 | 0.03443 | -2.60 | -1.70 | -1.300 | -0.750 | -0.1000 | 0.700 | 1.500 | 2.05000 | 3.9500 |
| 39 | 1821 | 0.01520 | -2.40 | -1.70 | -1.350 | -0.800 | -0.1000 | 0.650 | 1.500 | 2.02500 | 3.3000 |
| 40 | 2002 | 0.15069 | -2.30 | -1.60 | -1.300 | -0.700 | 0.0000 | 0.900 | 1.725 | 2.30000 | 4.0500 |
| 41 | 1299 | 0.21563 | -2.30 | -1.60 | -1.200 | -0.700 | 0.1000 | 0.925 | 1.750 | 2.40000 | 4.0000 |
| 42 | 414 | 0.32649 | -2.15 | -1.40 | -1.100 | -0.500 | 0.2000 | 1.000 | 2.000 | 2.70000 | 3.5000 |

Supplementary Table 2- Platelets concentration (10^9^/L ) percentiles according to gestational age

| **Gestational age at sample collection** | **Number of patients** | **Mean platelet number** | **P-1** | **P-5** | **P-10** | **P-25** | **P-50** | **P-75** | **P-90** | **P-95** | **P-99** |
| --- | --- | --- | --- | --- | --- | --- | --- | --- | --- | --- | --- |
| 0 | 328 | 273.1 | 125.0 | 174.0 | 188.0 | 226 | 264.0 | 312.5 | 372.0 | 398 | 457.0 |
| 1 | 500 | 279.6 | 150.5 | 178.5 | 196.0 | 228 | 268.0 | 320.0 | 368.5 | 408 | 513.5 |
| 2 | 575 | 267.5 | 123.0 | 169.0 | 188.0 | 219 | 260.0 | 306.0 | 366.0 | 390 | 437.0 |
| 3 | 705 | 265.0 | 105.0 | 170.0 | 192.0 | 222 | 260.0 | 305.0 | 349.0 | 385 | 444.0 |
| 4 | 895 | 273.2 | 139.0 | 173.0 | 196.0 | 228 | 264.0 | 312.0 | 362.0 | 401 | 469.0 |
| 5 | 1441 | 267.8 | 136.0 | 178.0 | 193.0 | 224 | 262.0 | 305.0 | 352.0 | 383 | 451.0 |
| 6 | 2181 | 260.7 | 132.0 | 169.0 | 186.0 | 218 | 255.0 | 297.0 | 339.0 | 373 | 438.0 |
| 7 | 2619 | 256.3 | 125.0 | 166.0 | 182.0 | 212 | 249.0 | 293.0 | 339.0 | 374 | 443.0 |
| 8 | 2934 | 256.3 | 130.0 | 165.0 | 184.0 | 213 | 251.0 | 293.0 | 336.0 | 367 | 430.0 |
| 9 | 2853 | 254.5 | 126.0 | 161.0 | 179.0 | 211 | 250.0 | 291.0 | 332.0 | 363 | 425.0 |
| 10 | 2806 | 250.3 | 133.0 | 164.0 | 181.0 | 208 | 245.0 | 288.0 | 329.0 | 353 | 414.0 |
| 11 | 2620 | 249.2 | 130.0 | 161.0 | 178.0 | 208 | 244.0 | 284.0 | 323.0 | 355 | 417.0 |
| 12 | 2673 | 246.6 | 133.0 | 164.0 | 177.0 | 207 | 243.0 | 280.0 | 318.0 | 346 | 413.0 |
| 13 | 2568 | 244.1 | 126.5 | 158.0 | 175.0 | 204 | 240.0 | 279.5 | 317.0 | 342 | 403.0 |
| 14 | 2506 | 243.5 | 123.0 | 156.0 | 173.0 | 204 | 239.5 | 278.0 | 317.0 | 343 | 408.0 |
| 15 | 2290 | 241.2 | 129.0 | 154.0 | 170.0 | 200 | 236.0 | 277.0 | 317.0 | 348 | 413.0 |
| 16 | 2380 | 241.3 | 126.0 | 156.0 | 172.0 | 201 | 237.0 | 276.0 | 317.0 | 344 | 400.0 |
| 17 | 3165 | 241.5 | 122.0 | 153.0 | 171.0 | 200 | 237.0 | 276.0 | 318.0 | 347 | 415.0 |
| 18 | 2818 | 241.6 | 121.0 | 155.0 | 171.0 | 201 | 237.0 | 277.0 | 319.0 | 347 | 395.0 |
| 19 | 2446 | 242.7 | 116.0 | 149.0 | 170.0 | 202 | 238.0 | 279.0 | 320.0 | 348 | 402.0 |
| 20 | 2060 | 243.4 | 119.0 | 155.5 | 173.0 | 202 | 239.0 | 280.0 | 319.0 | 345 | 402.0 |
| 21 | 1641 | 243.9 | 122.0 | 156.0 | 170.0 | 203 | 237.0 | 279.0 | 324.0 | 355 | 415.0 |
| 22 | 1529 | 245.2 | 124.0 | 155.0 | 172.0 | 200 | 238.0 | 282.0 | 330.0 | 362 | 429.0 |
| 23 | 1527 | 243.9 | 119.0 | 150.0 | 171.0 | 203 | 239.0 | 281.0 | 324.0 | 349 | 413.0 |
| 24 | 1593 | 244.5 | 115.0 | 152.0 | 168.0 | 202 | 238.0 | 280.0 | 327.0 | 355 | 420.0 |
| 25 | 1538 | 241.3 | 122.0 | 153.0 | 169.0 | 196 | 234.0 | 279.0 | 323.0 | 352 | 413.0 |
| 26 | 1468 | 241.1 | 124.0 | 150.0 | 168.5 | 196 | 233.0 | 279.0 | 324.0 | 356 | 421.0 |
| 27 | 1298 | 241.5 | 116.0 | 152.0 | 167.0 | 197 | 233.5 | 279.0 | 325.0 | 358 | 436.0 |
| 28 | 1139 | 241.8 | 112.0 | 149.0 | 166.0 | 197 | 234.0 | 276.0 | 329.0 | 359 | 429.0 |
| 29 | 965 | 239.2 | 121.0 | 149.0 | 165.0 | 194 | 232.0 | 276.0 | 323.0 | 358 | 419.0 |
| 30 | 864 | 239.7 | 110.0 | 147.0 | 165.0 | 194 | 231.0 | 279.0 | 322.0 | 354 | 426.0 |
| 31 | 801 | 239.2 | 113.0 | 145.0 | 165.0 | 196 | 234.0 | 277.0 | 321.0 | 352 | 421.0 |
| 32 | 757 | 232.7 | 114.0 | 141.0 | 159.0 | 189 | 225.0 | 271.0 | 323.0 | 345 | 406.0 |
| 33 | 714 | 234.7 | 114.0 | 138.0 | 156.0 | 188 | 226.5 | 276.0 | 319.0 | 349 | 433.5 |
| 34 | 779 | 229.4 | 108.0 | 139.0 | 155.0 | 182 | 222.0 | 264.0 | 314.0 | 349 | 437.0 |
| 35 | 799 | 233.2 | 111.0 | 139.0 | 159.0 | 185 | 223.0 | 272.0 | 323.0 | 354 | 448.0 |
| 36 | 1041 | 238.3 | 105.0 | 141.0 | 159.0 | 189 | 229.0 | 277.0 | 325.0 | 362 | 445.0 |
| 37 | 1887 | 236.4 | 113.5 | 140.0 | 156.0 | 189 | 226.0 | 274.0 | 329.0 | 363 | 461.0 |
| 38 | 3421 | 235.4 | 108.0 | 141.0 | 160.0 | 188 | 228.0 | 274.0 | 321.5 | 353 | 420.0 |
| 39 | 5552 | 233.3 | 113.0 | 140.0 | 157.0 | 187 | 226.0 | 270.0 | 320.0 | 352 | 431.0 |
| 40 | 7407 | 234.2 | 109.0 | 139.0 | 155.0 | 185 | 226.0 | 273.0 | 323.0 | 360 | 438.0 |
| 41 | 4685 | 228.3 | 104.0 | 137.0 | 153.0 | 183 | 219.0 | 264.5 | 316.0 | 348 | 416.0 |
| 42 | 1581 | 228.9 | 110.0 | 138.0 | 150.0 | 181 | 216.0 | 265.0 | 328.0 | 361 | 443.0 |

Supplementary Table 3- Fibrinogen concentration percentiles according to gestational age

| **Gestational age at sample collection** | **Number of patients** | **Mean fibrinogen concentration** | **P-1** | **P-5** | **P-10** | **P-25** | **P-50** | **P-75** | **P-90** | **P-95** | **P-99** |
| --- | --- | --- | --- | --- | --- | --- | --- | --- | --- | --- | --- |
| 0 | 20 | 450.4 | 287.0 | 288.0 | 301.5 | 356.5 | 429.5 | 464.5 | 742.5 | 799.5 | 808.0 |
| 1 | 23 | 423.0 | 218.0 | 225.0 | 260.0 | 301.0 | 353.0 | 448.0 | 714.0 | 836.0 | 1058.0 |
| 2 | 31 | 384.8 | 205.0 | 282.0 | 283.0 | 306.0 | 359.0 | 415.0 | 482.0 | 660.0 | 768.0 |
| 3 | 46 | 426.5 | 91.0 | 238.0 | 332.0 | 351.0 | 426.0 | 510.0 | 544.0 | 587.0 | 695.0 |
| 4 | 48 | 440.8 | 111.0 | 243.0 | 280.0 | 339.5 | 426.0 | 500.5 | 656.0 | 689.0 | 957.0 |
| 5 | 62 | 414.6 | 246.0 | 298.0 | 313.0 | 341.0 | 401.5 | 475.0 | 525.0 | 610.0 | 724.0 |
| 6 | 67 | 403.6 | 210.0 | 237.0 | 272.0 | 349.0 | 401.0 | 448.0 | 560.0 | 575.0 | 605.0 |
| 7 | 68 | 471.4 | 235.0 | 323.0 | 334.0 | 373.5 | 461.5 | 543.0 | 610.0 | 718.0 | 938.0 |
| 8 | 62 | 471.8 | 258.0 | 307.0 | 333.0 | 386.0 | 451.0 | 536.0 | 641.0 | 697.0 | 826.0 |
| 9 | 61 | 486.7 | 85.0 | 290.0 | 306.0 | 418.0 | 491.0 | 528.0 | 626.0 | 724.0 | 1012.0 |
| 10 | 72 | 471.8 | 243.0 | 346.0 | 384.0 | 404.5 | 448.0 | 514.5 | 578.0 | 637.0 | 1140.0 |
| 11 | 64 | 502.4 | 312.0 | 379.0 | 394.0 | 422.0 | 481.0 | 541.5 | 638.0 | 764.0 | 841.0 |
| 12 | 61 | 480.8 | 323.0 | 346.0 | 361.0 | 420.0 | 466.0 | 534.0 | 610.0 | 678.0 | 793.0 |
| 13 | 70 | 513.5 | 316.0 | 341.0 | 358.0 | 428.0 | 485.5 | 594.0 | 707.5 | 759.0 | 884.0 |
| 14 | 76 | 481.5 | 350.0 | 365.0 | 375.0 | 423.0 | 461.5 | 525.5 | 619.0 | 655.0 | 746.0 |
| 15 | 64 | 509.8 | 272.0 | 346.0 | 390.0 | 434.5 | 505.0 | 561.5 | 649.0 | 761.0 | 829.0 |
| 16 | 76 | 498.1 | 327.0 | 360.0 | 365.0 | 402.5 | 481.5 | 554.0 | 678.0 | 721.0 | 887.0 |
| 17 | 98 | 497.2 | 149.0 | 348.0 | 372.0 | 416.0 | 481.5 | 538.0 | 698.0 | 805.0 | 886.0 |
| 18 | 73 | 530.8 | 328.0 | 347.0 | 369.0 | 409.0 | 515.0 | 628.0 | 749.0 | 779.0 | 825.0 |
| 19 | 71 | 535.6 | 280.0 | 338.0 | 373.0 | 434.0 | 497.0 | 575.0 | 779.0 | 846.0 | 1303.0 |
| 20 | 66 | 500.1 | 285.0 | 349.0 | 360.0 | 409.0 | 480.0 | 562.0 | 663.0 | 763.0 | 923.0 |
| 21 | 94 | 514.4 | 207.0 | 330.0 | 382.0 | 436.0 | 519.0 | 592.0 | 654.0 | 740.0 | 851.0 |
| 22 | 112 | 519.6 | 283.0 | 354.0 | 391.0 | 437.0 | 479.0 | 606.0 | 701.0 | 794.0 | 828.0 |
| 23 | 179 | 519.2 | 201.0 | 321.0 | 359.0 | 421.0 | 506.0 | 607.0 | 689.0 | 766.0 | 932.0 |
| 24 | 166 | 502.2 | 281.0 | 346.0 | 364.0 | 412.0 | 485.0 | 582.0 | 634.0 | 696.5 | 897.0 |
| 25 | 177 | 521.8 | 263.0 | 364.0 | 391.0 | 436.0 | 503.0 | 588.0 | 685.0 | 755.0 | 905.0 |
| 26 | 198 | 525.9 | 227.0 | 327.0 | 370.0 | 453.0 | 520.5 | 589.0 | 685.0 | 757.0 | 901.0 |
| 27 | 256 | 531.8 | 305.0 | 335.0 | 399.0 | 439.0 | 521.0 | 606.0 | 684.0 | 772.0 | 863.0 |
| 28 | 246 | 542.2 | 251.0 | 345.0 | 382.0 | 447.0 | 519.5 | 610.0 | 720.0 | 825.0 | 1009.0 |
| 29 | 281 | 532.3 | 254.5 | 351.0 | 385.0 | 454.0 | 522.0 | 607.0 | 687.0 | 729.0 | 894.0 |
| 30 | 286 | 544.5 | 294.0 | 364.0 | 405.0 | 458.0 | 529.5 | 618.0 | 707.0 | 757.0 | 957.0 |
| 31 | 322 | 535.8 | 266.0 | 354.0 | 399.0 | 463.0 | 530.0 | 598.0 | 690.0 | 758.0 | 853.0 |
| 32 | 420 | 561.4 | 280.0 | 373.7 | 403.0 | 470.5 | 548.5 | 636.0 | 730.5 | 820.0 | 944.0 |
| 33 | 413 | 563.6 | 328.0 | 385.0 | 419.0 | 473.5 | 548.0 | 635.5 | 728.0 | 794.0 | 950.6 |
| 34 | 480 | 566.5 | 304.0 | 398.0 | 420.5 | 479.0 | 558.0 | 634.0 | 722.0 | 780.5 | 952.0 |
| 35 | 524 | 578.1 | 288.3 | 377.0 | 430.0 | 497.0 | 572.0 | 653.0 | 733.0 | 791.0 | 907.0 |
| 36 | 666 | 576.0 | 261.0 | 382.0 | 427.0 | 484.0 | 560.0 | 659.0 | 745.0 | 832.0 | 945.0 |
| 37 | 955 | 573.3 | 285.0 | 377.0 | 422.0 | 486.0 | 565.0 | 650.0 | 744.0 | 807.0 | 925.0 |
| 38 | 1257 | 568.8 | 281.0 | 369.0 | 419.0 | 488.0 | 557.0 | 636.0 | 734.0 | 804.0 | 953.0 |
| 39 | 1801 | 565.9 | 283.0 | 375.0 | 411.0 | 483.0 | 556.0 | 644.0 | 734.0 | 794.0 | 915.0 |
| 40 | 1995 | 547.8 | 282.0 | 360.0 | 398.0 | 467.5 | 541.0 | 622.0 | 702.0 | 762.3 | 880.0 |
| 41 | 1283 | 545.9 | 256.3 | 354.0 | 400.0 | 460.0 | 538.0 | 624.5 | 702.0 | 751.0 | 888.0 |
| 42 | 416 | 533.8 | 213.0 | 359.0 | 404.0 | 454.5 | 530.0 | 599.5 | 688.0 | 747.0 | 815.0 |

Supplementary information 1

Clinical definitions - Parity groups were defined as follows: multipara (2-5 deliveries) and grand-multipara (6 or more deliveries). Hypertension was defined in the presence of blood pressure ≥ 140/90 mmHg recorded in two separate measurements at least 4 hours apart. Mild hypertension was defined as a diastolic blood pressure ≥ 90 mmHg and <110 mmHg and/or systolic blood pressure ≥ 140 mmHg and < 160 mmHg. Severe hypertension was defined in the presence of diastolic blood pressure ≥110 and systolic blood pressure ≥ 160. Gestational hypertension was defined in the presence of hypertension developed after 20 weeks of gestation without proteinuria. Preeclampsia was diagnosed in the presence of elevated blood pressure and proteinuria of at least +1 in dipstick; its severity was defined according to the severity of hypertension and/or one of the following +3 proteinuria by dipstick, thrombocytopenia ≤ 100,000, elevated liver enzymes, persistent headache and/or blurred vision[1]. Gestational diabetes was diagnosed according to oral glucose tolerance test and were classified according to White’s classification[2]. Hydramnios - amniotic fluid index (AFI)>25 cm or when a vertical pocket of at least 8 cm was measured or as a subjective estimation of increased amniotic fluid volume. Oligohydramnios - AFI < 5 cm; a real time scanner equipped with a 3.5/5 MHz transducer of appropriate focal length estimated amniotic fluid volume. Preterm delivery was defined as- delivery before complete 37 weeks of gestation.

Newborns were classified, according to their weight, as: 1) Small for gestational age (SGA) - birthweight< the 10^th^ percentile, adequate for gestational age (AGA) birthweight from 10-90^th^ percentile, and large for gestational age (LGA)- birthweight> 90^th^ percentile according to regional growth curves[3]^.^. Prelabor rupture of membranes (PROM) was defined as rupture of the chorioamniotic membranes before the onset of labor. Uterine rupture was defined as a complete tear of the uterine wall including the visceral peritoneum with establishment of a direct communication between the uterine and abdominal cavities. Dehiscence - an opening of the previous cesarean scar with intact visceral peritoneum and no direct communication between the uterine and abdominal cavities. Post-partum fever - maternal temperature > 38°c that developed at least 24 hours after delivery recorded in two different measurements at least four hours apart or one measurement of maternal temperature of > 38.5°c regardless the time after delivery. Endometritis - postpartum maternal fever with clinical signs of tenderness above the uterine fundus or during cervical manipulation, foul vaginal discharge and positive endometrial culture. Wound infection was defined according to either clinical signs of infection or positive wound culture. Wound dehiscence - spontaneous opening of cesarean section wound including the abdominal fascia.

References

1. ACOG practice bulletin. (2002) Diagnosis and management of preeclampsia and eclampsia. Number 33. Obstet.Gynecol;99:159-67.

2 WHITE P. Pregnancy complicating diabetes.(1949) Am J Med;7:609-16.

3. Leiberman JR, Fraser D, Weitzman S, Glezerman M. (1993) Birthweight curves in southern Israel populations. Isr.J Med.Sci;29:198-203.
